# Epidemiological characteristics and predictors of late presentation of HIV infection in Barcelona (Spain) during the period 2001-2009

**DOI:** 10.1186/1742-6405-8-22

**Published:** 2011-07-06

**Authors:** Patricia Garcia de Olalla, Christian Manzardo, Maria A Sambeat, Inma Ocaña, Hernando Knobel, Victoria Humet, Pere Domingo, Esteve Ribera, Ana Guelar, Andres Marco, Maria J Belza, Josep M Miró, Joan A Caylà

**Affiliations:** 1 Epidemiology Service. Agencia de Salut Pública de Barcelona, Barcelona, Spain; 2 Consorcio de Investigación Biomédica en Red de Epidemiología y Salud Pública (CIBERESP; 3 Hospital Clinic-IDIBAPS. University of Barcelona, Barcelona, Spain; 4 Infeccious Diseases Unit, Hospital de la Santa Creu i Sant Pau. Universitat Autònoma de Barcelona, Barcelona, Spain; 5 Infectious Diseases, Hospital Vall de Hebron. Universitat Autònoma de Barcelona, Barcelona, Spain; 6 Internal Medicine-Infectious Diseases, Hospital del Mar, Barcelona, Spain; 7 Direcció General de Serveis Penitenciaris i de Rehabilitació. Departament de Justícia. Barcelona, Spain; 8 Escuela Nacional de Sanidad, Instituto de Salud Carlos III, Madrid, Spain

**Keywords:** HIV infection, Late presentation, Epidemiology, Predictors, Barcelona, Spain

## Abstract

**Background:**

Early diagnosis of HIV infection can prevent morbidity and mortality as well as reduce HIV transmission. The aim of the present study was to assess prevalence, describe trends and identify factors associated with late presentation of HIV infection in Barcelona (Spain) during the period 2001-09.

**Methods:**

Demographic and epidemiological characteristics of cases reported to the Barcelona HIV surveillance system were analysed. Late presentation was defined for individuals with a CD4 count below 350 cells/ml upon HIV diagnosis or diagnosis of AIDS within 3 months of HIV diagnosis. Multivariate logistic regression were used to identify predictors of late presentation.

**Results:**

Of the 2,938 newly diagnosed HIV-infected individuals, 2,507 (85,3%) had either a CD4 cell count or an AIDS diagnosis available. A total of 1,139 (55.6%) of the 2,507 studied cases over these nine years were late presenters varying from 48% among men who have sex with men to 70% among heterosexual men. The proportion of late presentation was 62.7% in 2001-2003, 51.9% in 2004-2005, 52.6% in 2006-2007 and 52.1% in 2008-2009. A decrease over time only was observed between 2001-2003 and 2004-2005 (p = 0.001) but remained constant thereafter (p = 0.9). Independent risk factors for late presentation were older age at diagnosis (p < 0.0001), use of injected drugs by men (p < 0.0001), being a heterosexual men (p < 0.0001), and being born in South America (p < 0.0001) or sub-Saharan Africa (p = 0.002).

**Conclusion:**

Late presentation of HIV is still too frequent in all transmission groups in spite of a strong commitment with HIV prevention in our city. It is necessary to develop interventions that increase HIV testing and facilitate earlier entry into HIV care.

## Introduction

In the European Union it is estimated that 15-38% of cases of HIV infection are diagnosed late [1] and 30% of infected individuals are not aware of their serological status, with proportions that vary between 12% and 20% in Sweden to more than 50% in Poland [2]. In Spain 56.3% of new diagnoses of HIV infection between 2003 and 2007 required treatment (CD4 < 350 cells/μl) at the time of diagnosis and 30.2% of these presented severe immunosuppression (CD4 < 200 cells/μl) [3].

Reducing the time elapsed between infection and the initiation of antiretroviral therapy (ART) is important to decrease progression of the infection and to facilitate immunological recovery. Therefore, early identification of infected individuals has been a priority of most AIDS prevention and control programs since the beginning of AIDS epidemic [4-6].

Delays in HIV care have serious public health implications because opportunities to prevent further transmission through effective ART are lost [7,8], and because initiating treatment for HIV infection at an advanced stage leads to poorer treatment outcomes than with early treatment [9,10]. Also, some studies have shown that after HIV infection diagnosis, most infected individuals remain sexually active, although most use safer practices, thereby limiting infection spread [11-13]. Late diagnosis of HIV also has economic implications for health services and society [14-16].

In Spain, all citizens enjoy universal free access to health services and ART is available for all patients. Delayed diagnosis and access to ART is essentially a public health problem and a loss of opportunity to limit the progression of disease and reduce the transmission of infection. For this reason, the objective of the present study was to determine the frequency of individuals presenting for care with a CD4 count below 350 cells/μl or presenting with an AIDS-defining event, to evaluate trends in this proportion, and to identify factors associated with late presentation of HIV infection in Barcelona during the period 2001-09.

## Methods

The city of Barcelona (1.6 million inhabitants), is located in the northern part of the east coast of Spain. In 2001, 11.89 AIDS cases and 17.47 cases of HIV infections were registered per 100,000 inhabitants. This figures decreased to 5.9 AIDS cases and 16.7 HIV infections in 2009. The Barcelona AIDS Surveillance System, which has been active since 1987, is an active system that collects data provided by doctors, hospital discharges, the tuberculosis (TB) register, and mortality databases regarding patients diagnosed with AIDS. The voluntary HIV Surveillance System, which has been active since 2001, collects information about new diagnoses of HIV infection in individuals older than 13 years tested in public or private facilities. All AIDS cases were collected through Barcelona AIDS Surveillance, and all HIV cases from the voluntary HIV Surveillance system.

Clinicians complete a standard data collection form and use a unique patient identifier code [17].

In this study, we considered all new diagnoses of HIV infection or of AIDS within 3 months of a positive HIV test among individuals older than 13 years who were resident in Barcelona during the period 2001-09. These cases of AIDS and HIV infection met the relevant criteria of the European Centre for AIDS/HIV Epidemiological Surveillance[18].

We analysed demographic data (sex, date and country of birth, year of arrival in Spain), HIV exposure category (injecting drug users [IDU], sex between men [MSM], heterosexuals [HT]), calendar period of HIV diagnosis (subdivided into 4 periods: 2001-03, 2004-05, 2006-07 and 2008-09), clinical data (AIDS, absolute CD4+ [CD4]).

According to the new European definition[19], late presenters (LP) was defined for persons presenting for care with a CD4 count below 350 cells/μl or presenting with an AIDS-defining event at the time of HIV diagnosis or within 3 months of the HIV-positive test, regardless of the CD4 cell count. Other patients were classified as non-late presenters.

### Statistical analyses

A descriptive analysis of the epidemiological characteristics of cases of HIV infection defined as late presenters was carried out.

For categorical and continuous variables, we compared the count (proportion) and median (interquartile range -IQR-), respectively, of late presenters to non late presenters. Categorical variables were compared using χ^2 ^test. For each comparison, the univariate odds ratio (OR), 95% confidence interval (CI) and p-value for statistical significance were computed. Continuous variables were compared using the Mann-Whitney U test or the Kruskal-Wallis test. Trends in LP over time were analysed using the χ^2 ^test for trend in proportions. Multivariate logistic regression was used to identify predictors of late presentation. A p-value of < 0.05 was considered to be statistically significant. Statistical analyses were performed using SPSS for Windows (Version 18.0; SPSS, Chicago, IL).

All data were collected by the HIV/AIDS Registry of Barcelona City and were handled in a strictly confidential manner according to the requirements of Spanish data protection Law[20].

## Results

During the study period, 2,938 new cases of HIV infection were detected, of which 2,268 (76.2%) were detected in hospital and 670 (22.8%) in non-hospital settings. The majority of cases were male (83.3%), with a median age of 35 years, MSM (51.8%) and 19.9% presented with AIDS. The most common AIDS-defining conditions were TB (26.2%), *Pneumocystis jirovecii *pneumonia (24.3%) and Kaposi Sarcoma (10.3%). In 90% of cases the AIDS diagnosis was made within 30-days of HIV infection. We observed that the proportion of IDU among males decreased during the study period (p < 0.0001), whereas the proportion of MSM increased from 40.2% in 2001-03 to 61.9% in 2008-09 (p < 0.0001). However, no significant trend was observed in IDU and HT women (p = 0.1 y p = 0.2, respectively) (Table 1). 55% of cases were born in Spain. The proportion of migrants increased from 22.6% in 2001-03 to 45.8% in 2008-09 (p < 0.0001), of which 37.8% had been resident in Spain for less than 1 year, 38.8% between 2 and 5 years and 8.3% more than 10 years.

**Table 1:** Characteristics of newly diagnosed HIV-infected patients in Barcelona (Spain), 2001-2009

Characteristics	2001-2003;n = 956 (100%)	2004-2005;n = 630 (100%)	2006-2007;n = 691 (100%)	2008-2009; n = 661 (100%)	Total; N = 2938 (100%)
Sex					
Male	768 (80.3)	502 (79.7)	599 (86.7)	581 (87.9)	2450 (83.4)
Female	188 (19.3)	128 (20.3)	92 (13.3)	80 (12.1)	488 (16.6)

Age at HIV diagnosis*					
All	35.1 (29.8-42.9)	34.7 (28.9-41.7)	35.5 (29.9-43.1)	34.3 (29.2-41.1)	35.0 (29.4-42.2)
Male	35.2 (29.8-42.8)	34.8 (29.3-41.6)	35.5 (29.8-42.7)	34.2 (29.0-40.8)	35.1 (29.5-35.1)
Female	34.5 (29.8-42.9)	36.7 (27.4-42.1)	35.9 (30.0-44.9)	35.3 (29.3-44.8)	34.5 (29.3-43.4)

Region of birth					
Spain	551 (57.6)	353 (56.0)	368 (53.3)	344 (52.0)	1616 (55.0)
Western Europe & North America	53 (5.5)	30 (4.8)	53 (7.7)	54 (8.2)	190 (6.5)
Latin America & Caribbean	147 (15.4)	133 (21.1)	191 (27.6)	178 (26.9)	649 (22.1)
Middle East & North Africa	22 (2.3)	7 (1.1)	12(1.7)	9(1.4)	50(1.7)
Sub-Saharan Africa	30 (3.1)	33 (5.2)	20 (2.9)	20 (3.0)	103 (3.5)
Eastern Europe	14 (1.5)	12 (1.9)	20 (2.9)	36 (5.4)	82 (2.8)
Asia	6 (0.6)	6 (1.0)	1 (0.1)	6 (0.9)	19 (0.6)
unknown	133 (13.9)	56 (8.9)	26 (3.8)	14 (2.1)	229 (7.8)

Exposure category by sex**					
IDU men	181 (18.9)	65 (10.3)	52 (7.5)	29 (4.4)	327 (11.1)
MSM	384 (40.2)	320 (50.8)	410 (59.3)	409 (61.9)	1523 (51.8)
HT men	149 (15.6)	85 (13.5)	93 (13.5)	87 (13.2)	414 (14.1)
IDU women	48 (5.0)	22 (3.5)	10 (1.4)	12 (1.8)	92 (3.1)
HT women	129 (13.5)	102 (16.2)	74 (10.7)	54 (8.2)	359 (12.2)
unknown	65 (6.8)	36 (5.7)	52 (7.5)	70 (10.6)	223 (7.6)

CD4 cell count and AIDS					
AIDS	241 (25.2)	105 (16.7)	138 (20.0)	100 (15.1)	584 (19.9)
< 200	141 (14.7)	78 (12.4)	81 (11.7)	74 (11.2)	374 (12.7)
200-349	123 (12.9)	96 (15,2)	100 (14,5)	116 (17.5)	435 (14.8)
350-499	111 (11,6)	92 (14.6)	117 (16.9)	108 (16.3)	428 (14.6)
500 and more	189 (19.8)	167 (26.5)	171 (24.7)	159 (24.1)	686 (23.3)
unknown (%)	151 (15.8)	92 (14.6)	84 (12.2)	104 (15.7)	431 (14.7)

*Median (interquartile range). **IDU: injection drug use; MSM: men who have sex with men; HT: heterosexual contact

Either a CD4 cell count or an AIDS diagnosis were available for 85.3% (2,507) of the 2,938 newly diagnosed HIV-infected individuals. Patients whose CD4 cell count was not available differed from those for whom CD4 count was available in the frequency of IDU (26.0% *vs *12.9%, p < 0.001) and in the median age (34.43 years *vs *37.39 years, p < 0.001).

**Figure 1:**
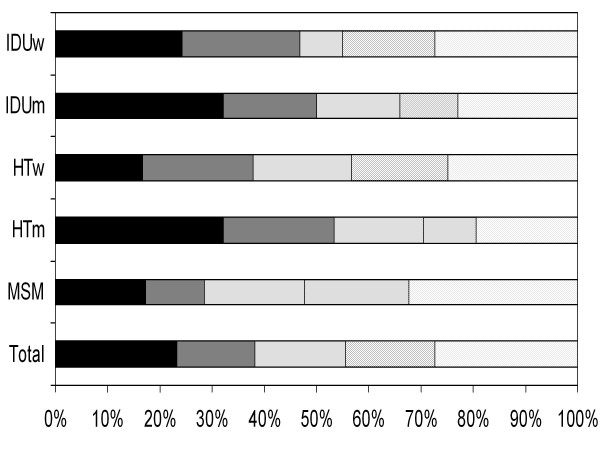
**Proportion of individual by CD4 count/AIDS at the time of HIV diagnosis according to sex and category of exposure in Barcelona (Spain): 2001-2009**. IDUw: injection drug use women; IDUm: injection drug use men; HTw: heterosexual women; HTm: heterosexual men; MSM: men who have sex with men.

The proportion of patients who were LP was 55.6% (1,393 persons) and 38.5% had advanced HIV infection (15.2% presenting with CD4 < 200 cell//μl and 23.3% with AIDS regardless of the CD4 cell count). A decrease of LP over time only was observed from 62.7% in 2001-03 to 51,9% in 2004-05 (p = 0.001), but remained constant thereafter (p = 0.9). Figure 1 shows the proportion of individual by CD4 count/AIDS at the time of HIV diagnosis according to category of exposure: 48% of MSM had < 350 CD4/μl, whereas among IDU men and HT men this percentage was 66% and 70%, respectively.

LP was slightly, but not significantly, more frequent among women than among men (58.5% *vs *55.0%). However, differences were observed according to age, region of birth, category of exposure and period of diagnosis (Table 2). The logistic regression model showed that risk of LP was associated with age: for each increment of 5 years, risk of being a late presenter increased by 38%; with region of birth: LP was significantly more common among individuals born in Latin America and Caribbean (OR:1.48; CI: 1.20-1.83) and Sub-Saharan Africa (OR:2.43; CI: 1.14-4.17) than among individuals born in Spain; with category of exposure and sex: IDU and HT men showed an OR for risk of being LP of 2.12 (1.57-2.87) and 1.79 (1.38-2.34), respectively, compared to MSM. A decrease in the risk of being LP was observed in the last three periods compared to the period 2001-03 (Table 2).

**Table 2:** Characteristics and factors associated with late presentation for newly HIV diagnosis in Barcelona (Spain), 2001-2009

Characteristics	Total N = 2507	Late presenters (%)	Unadjusted OR (CI)	p value	Adjusted OR (CI)	p value
Sex						
Male	2097	1153 (55.0)	1	0.18		
Female	410	240 (58.5)	1.16 (0.93-1.44)			

Age at HIV diagnosis*						
All	35.4 (29.6-42.8)	37.9 (31.5-46.2)		0.0001	1.38 (1.27-1.40)	<0.0001
Male	35.4 (29.7-42.5)	38.0 (31.8-45.8)				
Female	35.3 (29.4-44.4)	37.7 (30.6-47.2)				
Age (per a 5 yrs increase)						

Region of birth						
Spain	1460	826 (56.6)	1	0.002	1	0.279
Western Europe & North America	166	73 (44.0)	0.60 (0.43-0.84)	0.2	0.83 (0.59-1.16)	< 0.0001
Latin America & Caribbean	570	315 (55.3)	0.95 (0.78-1.16)	0.8	1.48 (1.20-1.83)	0.838
Middle East & North Africa	44	26 (59.1)	1.11 (0.58-2.13)	0.02	1.07 (0.56-2.03)	0.002
Sub-Saharan Africa	74	52 (70.3)	1.81 (1.08-3.12)	0.9	2.43 (1.14-4.17)	0.274
Eastern Europe	64	36 (56.3)	0.99 (0.58-1.68)	0.2	1.35 (0.79-2.31)	0.219
Asia	18	13 (72.2)	2.00 (0.66-6.44)	0.05	1.96 (0.67-5.74)	0.107
unknown	111	52 (46.8)	0.68 (0.45-1.01)		0.71 (0.47-1.07)	

Exposure category**						
MSM	1333	636 (47.7)	1	< 0.001	1	< 0.0001
IDU men	252	166 (65.9)	2.12 (1.58-2.83)	< 0.001	2.12 (1.57-2.87)	< 0.0001
HT men	382	269 (70.4)	2.61 (2.03-3.36)	0.9	1.79 (1.38-2.34)	0.340
IDU women	62	34 (54.8)	1.33 (0.78-2.29)	0.004	1.29 (0.76-2.20)	0.279
HT women	319	181 (56.7)	1.44 (1.12-1.85)	< 0.001	1.16 (0.88-1.51)	0.009
unknown	159	107 (67.3)	2.26 (1.57-3.24)		1.63 (1.13-2.37)	

Year of diagnosis						
2001-2003	805	505 (62.7)	1	< 0.001	1	0.001
2004-2005	538	279 (51.2)	0.64 (0.51-0.80)	< 0.001	0.67 (0.53-0.85)	0.001
2006-2007	607	319 (52.6)	0.66 (0.53-0.82)	< 0.001	0.67 (0.53-0.85)	0.001
2008-2009	557	290 (52.1)	0.65 (0.52-0.81)		0.71 (0.56-0.90)	

*Median (interquartile range). **IDU: injection drug use; MSM:men who have sex with men; HT:heterosexual contact

In 650 (33.8%) cases of the 1,923 new diagnoses of HIV infection with CD4 count data and without AIDS the median time elapsed between the diagnosis of infection and the first determination of CD4 was 56 days (15-129), with differences according to category of exposure. Among IDU, the median time interval was 162 days (40-495), while for other exposures it was 54 days (14-115) (p = 0,002).

## Discussion

This study shows that a high proportion of individuals (55.6%) meet the criteria of the new European definition of LP (CD4 < 350 cells/μl or AIDS). Although it is difficult to obtain data on LP in large cities, these figures do not differ greatly from those observed at national or regional level, even in areas where the prevention of HIV infection is a priority [1,3,21,22]. Thus, 50% of new diagnoses of HIV infection occur when the subject needs treatment [23]. If we take into account the new ARV treatment recommendations, which indicate that treatment should be started in asymptomatic patients with CD4 < 500 cells/μl, this proportion reaches 70% [24-26]. These high proportions of patients with *"care-stage" *HIV infection at their initial clinic visit suggests that barriers to HIV care are also considerable in developed countries. The negative impact of LP is a likely increase in morbidity and mortality [27,28], more potential transmission at the community level [13] and further treatment costs [14-16].

The proportion of LP decreased only between 2001-03 and 2004-05, as has been observed in other studies [3,29], it continues to be high in all groups. LP is associated with age and affects all subpopulations, although its distribution is not homogeneous [3,30]^. ^The limited perception of risk [31] compounds the fact that tests tend to be offered less frequently to older individuals. Heterosexual individuals over 50 years are not the target of most prevention campaigns [32]. In general, individuals born outside Spain presented greater risk of LP than Spanish nationals, most notably individuals born in Latin America and Sub-Saharan Africa, as observed in other studies [3,10]. In a study carried out in Italy, a greater proportion of LP was also observed in the immigrant population [29].

In relation to sex and the mechanism of transmission, it was observed that IDU men and HT men presented a higher frequency of LP compared to MSM. This higher proportion of delayed diagnoses among heterosexuals compared to cases of homosexual transmission has been highlighted in various studies [33-35]. A greater probability of delayed diagnosis has been observed in Italy, but only in drug addicts that remained isolated from social and health services [36]. However in our study, comparing the MSM group with IDU women or HT women this difference disappears. This may be due to women's higher perception of risk, their greater likelihood of attending health services [37] and the probability that they will be offered the test during pregnancy or birth, as a result of which they will be diagnosed earlier than HT or IDU men.

Some results of this study suggest that the delay among IDU is more a delay in beginning treatment than in diagnosis of the infection, since they are often diagnosed in drug rehabilitation centres or in prison. In this sense, the overrepresentation of IDU without lymphocyte count data would indicate that the diagnostic test is carried out on these individuals, but many are lost to follow-up. Moreover, the median time elapsed between the diagnosis of infection and the determination of CD4 was notably higher among the IDU (162 days) compared to other categories of exposure (54 days), which would indicate that the delay in this group was more likely due to a delay in beginning treatment than delayed diagnosis, as observed in other studies [38,39]. Consequently, public health strategies aimed at ensuring access to health resources are a priority for diminishing LP in this group. An early diagnosis, especially in this group does not necessarily require that the patient be undergoing follow-up or treatment [40,41]. However, reinforcing the strategies that increase the availability of the test should be directed in the first place at those individuals likely to be at high risk for HIV infection based on the presence of indicator situations or events where the estimated prevalence of HIV-1 infection is above 1%, and at the most vulnerable groups [42-44].

Among the limitations of this study, we highlight the number of cases without data on lymphocyte count (14.7%) with a different distribution in the case of the IDU, which may lead to underestimation of the results for this group in our study. Another limitation could be the under-reporting of HIV infection due to the fact that this was voluntary. This underreporting is estimated around 15%-20% (unpublished data).

## Conclusion

In conclusion, late presentation of HIV is common in spite of a strong commitment to universal access to HIV infection prevention, diagnosis and treatment in our city. This study reveals a need to develop interventions that increase HIV testing and facilitate earlier entry into care, such as routine screening in healthcare and non-clinical settings for patients at risk for HIV (e.g. have unprotected sex with many sex partners, have a sexually transmitted infection, have a diagnosis of tuberculosis, use illegal injected drugs, pregnancy). An accurate sexual history and other risk factors, together with knowledge of country of birth, can identify most individuals who should be offered an HIV test and a link to care.

## Abbreviations

ART: antiretroviral therapy; CI: confidence interval; HIV: human immunodeficiency virus; HT: heterosexual relationships; HTw: heterosexual women; HTm: heterosexual men; IDU: injecting drug users; IDUw: injection drug use women; IDUm: injection drug use men; late presenters (LP): persons presenting for care with a CD4 count below 350 cells/ml or presenting with an AIDS-defining event at the time of HIV diagnosis or within 3 months of the HIV-positive test, regardless of the CD4 cell count; MSM: sex between men; OR: odds ratio; TB: tuberculosis.

## Competing interests

The authors declare that they have not competing interests.

## Authors' contributions

PGdeO, JAC designed the study, interpreted the data, and drafted the manuscript. PGdeO and MJB analysed the data. CM, MAS, IO, HK, VH, PD, ER, AG, AM, JMM and the HIV Surveillance Group collected data and notified the cases, and participated in writing and revised critically the manuscript. All authors have reviewed and approved the final manuscript.
